# Medicaid managed care organizations' experiences with network adequacy

**DOI:** 10.1093/haschl/qxaf049

**Published:** 2025-03-13

**Authors:** Jane M Zhu, Ruth Rowland, Daniel Polsky, Inga Suneson, Simon F Haeder, Deborah J Cohen, K John McConnell

**Affiliations:** Division of General Internal Medicine, Oregon Health & Science University, Portland, OR 97239, United States; Center for Health Systems Effectiveness, Oregon Health & Science University, Portland, OR 97239, United States; Carey School of Business, Johns Hopkins University, Baltimore, MD 21202, United States; Center for Health Systems Effectiveness, Oregon Health & Science University, Portland, OR 97239, United States; Department of Health Policy and Management, Texas A&M University, College Station, TX 88843, United States; Department of Family Medicine, Oregon Health & Science University, Portland, OR 97239, United States; Center for Health Systems Effectiveness, Oregon Health & Science University, Portland, OR 97239, United States

**Keywords:** behavioral health, Medicaid, managed care, provider networks, workforce

## Abstract

Access to behavioral health care continues to be a challenge in Medicaid, where most enrollees are restricted to networks of providers and facilities contracted with managed care organizations (MCOs). While state and federal regulations have sought to ensure access to care, little is known about how health plans perceive and respond to these network adequacy standards. We interviewed 27 administrators and executives across 19 local, regional, and national Medicaid MCOs to assess their behavioral health networks and perceived barriers and facilitators in these efforts. We purposively sampled MCOs for maximum heterogeneity, with early findings used to refine subsequent recruitment targets until thematic saturation. We used an iterative inductive coding approach with code discrepancies analyzed and reconciled until consensus was reached. Five major themes arose: existing regulations often failed to capture true access gaps; MCOs used supplementary approaches to monitor network adequacy; limited corrective actions were available; access measures were more meaningful when grounded in enrollee experiences; and provider directory accuracy was challenged by logistical barriers. In this first study to examine MCOs' experiences with network adequacy monitoring, our findings suggest key deficiencies with current regulations and opportunities to support MCOs more broadly as policymakers seek to strengthen network adequacy regulations.

## Introduction

Medicaid is the single largest payer for behavioral health services (ie, mental health and substance use disorder treatment) in the United States. More than 3 in every 10 enrollees have a diagnosed mental health condition, and many enrollees report inadequate access to care.^1^ For example, as of 2018, half of Medicaid enrollees with serious mental illnesses like psychosis or affective disorders reported unmet mental health care needs.^2^ An important lever by which such access challenges can be alleviated—or exacerbated—is through Medicaid managed care. Nearly 80% of Medicaid enrollees are now covered by managed care organizations (MCOs), which are responsible for constructing networks of providers and facilities to deliver care to their enrollees. Behavioral health networks may include specialty prescribers (eg, psychiatrists and psychiatric mental health nurse practitioners), psychotherapists and counselors, behavioral health treatment facilities, and programs like assertive community treatment.

Both “push” and “pull” factors influence the construction of managed care behavioral health networks. Supply-side constraints, including persistently low acceptance of insurance among psychiatrists and other mental health providers,^3-8^ are particularly pronounced in Medicaid.^9^ These low rates of insurance acceptance have been attributed to substantial reimbursement disparities, administrative burdens associated with Medicaid billing and claims processing, and insufficient workplace resources to adequately support enrollees' medical and social complexity.^10-16^ MCOs may also selectively contract with providers in order to purposefully contain costs and/or oversee quality of care.^17,18^ Studies in other insurance markets, for instance, show that narrow network plans are associated with lower premiums than plans with broader networks, without clear reductions in quality of care.^17^

There is evidence, however, that narrow behavioral health networks restrict access to care. Mental health networks are narrower than primary care and other specialty networks,^18,19^ and approximately 40% of psychiatrist networks in Medicaid managed care contain less than a quarter of all psychiatrists in a given market.^3^ In turn, inadequate networks have been associated with reduced access to specialists,^20,21^ with delayed or foregone mental health treatment.^20,22^ Furthermore, narrow networks are challenging to identify and measure, as regulatory and consumer-facing directories of in-network providers are frequently out-of-date with high rates of inaccuracies.^23-25^ In this setting, MCOs can directly affect access to mental health services through contracting practices, responses to performance metrics, or deployment of resources to address network gaps.

Thus, state and federal agencies have increasingly turned to network adequacy standards, regulating that MCOs must deliver services to enrollees in a timely and sufficient manner. Since 2020, Medicaid managed care plans have been required to adopt at least one quantitative standard, such as maximum travel distance or time, minimum provider ratios, or maximum wait times, to assess network adequacy. There is a growing need to understand how these health plans perceive and respond to existing network adequacy standards. To explore how Medicaid MCOs assess the adequacy of their behavioral health networks and perceived barriers and facilitators in these efforts, we conducted interviews with 27 administrators and executives across 19 local, regional, and national Medicaid MCOs. Findings from this work are intended to inform ongoing MCO approaches as well as state and federal regulations to ensure appropriate access to services.

## Methods

### Setting, participants, and study design

We identified Medicaid MCOs across all U.S. states using Kaiser Family Foundation's Medicaid managed care tracker data^26^ enhanced with Medicaid enrollment files^27^ and state-based web searches. We constructed a sampling matrix based on MCO characteristics (eg, for-profit status, national carrier vs. regional or local health plan, size of enrollee population), as well as state characteristics (eg, behavioral health carve-in vs. carve-out financing structure, Medicaid expansion status, and number of MCOs in a state). States with fewer than 3 MCOs were excluded to ensure de-identification of MCOs and participants.

We used this sampling matrix to purposively sample MCOs for maximum heterogeneity, following best practices for sampling in qualitative research.^28^ We recruited at least one key informant from each MCO, including administrative or operational leadership who directly engaged with or oversaw provider network development, recruitment, contracting, credentialing, maintenance, and quality activities for behavioral health. We contacted a total of 49 MCOs, with recruitment continuing in stages until saturation was reached (ie, at 27 interviews).^29^ Oregon Health & Science University's Institutional Review Board approved this study.

### Data collection

Three members of the research team conducted semi-structured video interviews from October 2023 through June 2024, each lasting approximately one hour via a remote teleconferencing platform. Interviews were conducted with one or more MCO leaders from each MCO. Some participants preferred to have other members of their team present, and in these cases, interviews included between 2 and 4 participants. Early findings were used to refine subsequent recruitment targets and monitoring for saturation, with data collection occurring in parallel with analysis. Recruitment ceased when additional data did not lead to any new emergent themes or new information being generated in line with thematic saturation.^30^ While compensation for participation was offered, no participants accepted payment for their time.

The study team initially developed the interview protocol based on pilot testing with feedback from interviewees and iteratively refined it with subsequent interviews. Interview guides were developed separately for locally and nationally operating MCOs. Domains included approaches to developing, maintaining, and expanding behavioral health provider networks, responses to and perceptions of state and federal network adequacy regulations, internal MCO processes for monitoring and assessing network adequacy, and approaches to provider directory maintenance and validation.

## Data analysis

Interviews were recorded with permission and professionally transcribed, with transcripts deidentified and verified for accuracy. Team members with qualitative methods expertise began data analysis by inductively coding the first 8 interviews, using an iterative approach to develop a coding scheme based on recurring themes. Codes were refined continuously as more data was collected, and the revised list of codes was applied to all previously coded transcripts. Code discrepancies were analyzed and reconciled until consensus was reached, with remaining transcripts coded independently. Team members discussed analytical questions iteratively to identify preliminary findings and implications from the analysis, and to bundle codes into major themes. We used Atlas.ti (version 23.3.0; ATLAS.ti Scientific Software Development, GmBH) for data management and analysis.

## Limitations

A key limitation of this study is that we did not interview state or federal regulators who may perceive network adequacy efforts differently from those entities being regulated. While we attempted to maximize sampling heterogeneity, purposive sampling may preclude transferability of results to specific MCOs or states. Finally, in this qualitative study, we are unable to determine whether MCOs' reported approaches to provider network monitoring and interventions are associated with improved behavioral health care access and outcomes.

## Results

The 19 MCOs interviewed (response rate 38.8%) included 14 (87.5%) single-state community health plans and 2 (12.5%) state-level subsidiaries of national for-profit carriers (Table 1). An additional 3 interviews were with leadership teams of national carriers (1 not-for-profit, 2 for-profit) with combined operations in more than 30 states. Analyses revealed five major themes pertaining to network adequacy monitoring and assessment (Table 2).

**Table 1.** MCO characteristics.

MCO	# of interviews (# of participants)	US region	BH carve-out state	Populations covered	Profit status	Health system affiliated or owned	Coverage (single state vs multistate)
**1**	1 (1)	West		Comprehensive	Non-profit		Single state
**2**	1 (1)	South	Yes	Physical health + mild/moderate BH only	Non-profit	Yes	Single state
**3**	1 (1)	South		Comprehensive	Non-profit		Single state
**4**	1 (1)	West		Comprehensive	Non-profit		Single state
**5**	1 (1)	Midwest	Yes	Physical health + mild/moderate BH only	Non-profit		Single state
**6**	1 (1)	Midwest		Comprehensive	Non-profit	Yes	Single state
**7**	1 (1)	West		Comprehensive	Non-profit		Single state
**8**	1 (1)	West	Yes	BH only	Non-profit		Single state
**9**	2 (2)	West	Yes	Physical health + mild/moderate BH only	Non-profit	Yes	Single state
**10**	1 (1)	Midwest		Comprehensive	Non-profit		Single state
**11**	1 (1)	Northeast		Comprehensive for dual-eligible	Non-profit		Single state
**12**	1 (1)	Midwest	Yes	Physical only	For-profit		Single state
**13**	1 (3)	Northeast	Yes	BH only	Non-profit	Yes	Single state
**14**	2 (2)	South	Yes	Comprehensive for SMI population	Non-profit		Single state
**15**	1 (1)	West		Comprehensive	For-profit		Single state
**16**	1 (1)	South		Comprehensive for SMI population	Non-profit		Single state
**17**	1 (4)	National		Comprehensive	For-profit		Multistate
**18**	1 (1)	National		Comprehensive	Non-profit	Yes	Multistate
**19**	1 (2)	National		Comprehensive	For-profit		Multistate
**Total**	21 (27)						

Data from authors' interviews of Medicaid MCOs in the U.S.

BH, behavioral health.

**Table 2.** Selected quotes.

Theme	Selected quotes
**Challenges with existing network adequacy requirements**	*[The] number of clinicians … and how far away are they … there's so many nuances underneath that … that we really feel are important. So I don't know that it really informs a lot of what we do. We always are exceeding the state and federal standards, but I wouldn't say that means we have an adequate network*.
	*On paper everything looks perfect. But you start hearing the stories of people and how they can't make an appointment or they can't get in for 60 days.*
	*You have [to have] access to a provider within 30 min or 30 miles. Those don't mean a whole lot to me if I'm being completely honest, having a provider within 30 min or 30 miles of where you live is not accessible care to me. If I had to drive 30 min to see a therapist, I would not go see a therapist.*
	*We don't limit adding any providers through our Medicaid network. We want as many providers as possible. I just think it's the volume of people who are in need in comparison to what really is out there … We have 140 000 members and [another MCO] has 150 000 members… and they're all using the same providers so there is going to be an issue.*
**Internal approaches to monitoring network adequacy**	*We're recognizing that even monthly [tracking] is not adequate, it's much more fluid than that. So we're looking at other models of representing that and having a much stronger connectivity. And it's likely to be a digital connectivity with our network …We have a few of these larger providers who will frequently send us messages, “Hey, I've got these openings and I can handle this.” And we provide that [data] to our case management team and our members services team.*
	*It's usually through [case management and utilization management] teams that we get made aware of, “Oh my God, the wait lists for this service are bananas right now. We need more of this type of provider. This provider closed its doors.” Those providers are the ones that get thrown to the top of the list for recruitment efforts or all kinds of creative problem solving*.”
	*We’ve dedicated a fair amount of resources to thinking about capacity, availability and utilization… Everything from numbers of providers, ratios to members, appointment wait times to member satisfaction surveys. That allows us to then tailor interventions … thinking of ways in which we support members. People routinely call our care management and customer service lines when they're having problems finding a provider. And we'll do facilitated connections, but we also have broader approaches where we think about [deficits] in conjunction with our county partners, procurement, expansion, rate adequacy, program innovation, different approaches to make sure that there's adequate capacity.*
**Limited corrective actions available**	*Unfortunately the tools that we have to work with are pretty limited. It tends to be dollars, and we could dangle as many dollars out there as we had. There's some places where it's just going to continue to be a perennial challenge. So then it is about how do we upskill our primary care network, how do we use things like Project Echo or telephonic services or others to try and expand the scope of access [despite] what's geographically available in that region.*
	*Right now, there are fines. If we don't meet network adequacy, we have to have a corrective action plan, but we can also have liquidated damages. So, it's a punitive system, and there's not the incentive to give that accurate answer. Let's look with humility at our network and say, “Where are the gaps? Where do we need to improve?” There's an area right now … I don't have to report it as a gap, but we're so close, we're fragile. If one provider were to leave, all of a sudden we're not doing well. But we don't really ask.*
	*I'm not certain I agree with the state's requirement where it's a six-month period, and they're off your network [if you don’t see a Medicaid patient]. People may have gone on maternity leave … all kinds of things. We shouldn't make providers start all over from scratch to get back in-network. If we're going to make it easier for providers to work in the Medicaid business, we have to keep them engaged. We can't kick them out just because they haven't seen a client in six months.*
	*If we access the network, and we realize that if you've got a ratio of 1-to-150, and you don't even have 100 providers in that particular area, it's a rather easy decision to not go into that market.*
**Meaningful network adequacy assessments**	*We pay a lot of attention to rising risk. When we see higher demand for things like inpatient and residential treatment for our kids, we know we need more subacute services to help keep kids out of residential services. We have a really robust clinical team that pays attention to those things … far more valuable indicators of access than whether somebody has a provider within 30 min or miles*.
	*It would be helpful just to survey members. What do they need? What do they really want? … It's good to have at least those [time and distance] numbers, but I really need to understand … the member's perspective.*
	*There's the superficial structural of, “Do we have enough providers?” Then there's performance of the network and accessibility for limited English proficiency, or hard of hearing, blind, etc. … Those are things we aspire to.”*
	*We need to start looking at accreditations. If we have providers who are accredited and are held to these standards and audited by those accreditation agencies, we need to somehow look at how the two tie together from what the state wants to glean [regarding network adequacy], and combine those two and not create [more burden] for providers.*
	*One of the biggest gaps over the years, has been [how members] navigate the system. I'd start there and say, “How do you define network adequacy? What is it you've found that was adequate that wasn't, what does that mean?” Sometimes it's getting into a service, but it's also staying in the service. I've got providers that I know I can get someone into, but I don't know if they'll be there three months from now. I would love it if someone at a higher level grounded this in the experience of people with mental illness and their families.*
**Challenges with provider directory accuracy**	*If we don't receive notification from someone that they decided to move their clinic … we can't update it. That's the biggest issue … just notification and knowing. In our provider contracts, we do say, you need to notify us within 30 days so we can update our directories. It doesn't always happen.*
	*It's near impossible to keep them current. Providers that move offices, they don't tell us. But some of it is … there's organizations that we contract with, and then there's all these different individual providers underneath each organization. As a patient, if I'm trying to find a new physician, I [care about] “Can I go to this office?” But that's not how a lot of directories work. You need to look up every single doctor within the practice. It doesn't make for a very user-friendly experience when the data is organized in that way.*
	*CMS is coming out with tighter guidelines as far as updates and all that. We're working hard on that to make sure we can meet it. But when we don't have the information, it's just really hard if people don't tell you. That's why we have to audit. Some of those audits include calling the provider … to make sure that we have the right information.*
	*The plan absolutely makes mistakes, whether it's manual entry or something happens with the logic script and it doesn't run or whatever. But there are so many times where I get an audit back from the state or from CMS, and it's got that list of errors … and on the document that we got from the provider, it said that [error].*
	*Our utilization management department, our care managers, they will have their own list to say, “If our members need help, we know these are the counselors that we should refer them to for addiction therapy. These are the counselors for anxiety and depression.” But we don't really do a great job of putting that back into our system to help members who are just naturally looking on their own and who maybe aren't getting the services of our care management team.*

Data from authors' interviews of Medicaid MCOs in the U.S.

### Theme 1: MCOs questioned how well existing network adequacy regulations captured access gaps

MCOs reported facing varying requirements to report network adequacy data to state regulators, including highly detailed documentation requirements submitted as frequently as monthly. Some MCOs also received reports bi-directionally and were asked to address deficiencies that the state identified. Participants viewed these requirements differently: while some participants called these efforts “symbolic” in nature, others perceived these deliverables to be important minimal requirements to ensure consistent attention to access issues.

Participants also questioned how well existing adequacy requirements captured true access gaps. While plans generally reported meeting or exceeding state and federal network adequacy standards, participants highlighted deficiencies in existing geospatial measures, like time and distance standards, as well as static measures, like provider counts, which failed to capture known gaps within their networks (Table 2). MCOs believed that several aspects of care that were important to member experience, including wait times, provider quality, and service alignment with care needs, were overlooked: “*It's very time-consuming, very resource intensive to do what we do now. But … we're giving highly precise, inaccurate information to the wrong questions.”*

MCOs particularly questioned the ability of existing adequacy measures to account for the dynamic nature of provider networks resulting from constant turnover. As a Vice President of Medicaid programs noted: “*Part of the challenge is that … you might have access one month. Then, if you lose a particular practitioner the next month, we may not know about that until it catches up with us because we don't have a real-time way of knowing what is happening in any clinic*.” MCOs also reported little visibility into provider capacity and availability, particularly in overlapping coverage areas where competing plans often relied on the same sets of providers.

### Theme 2: MCOs employed supplementary approaches to monitoring network adequacy

While some MCOs contracted with third-party contractors to assess network adequacy, others conducted network assessments internally, often relying on individuals or small teams to manually track contracted providers, monitor member grievances, and consult with clinical, care management, and utilization management teams to evaluate access gaps. As one director of network management said: “*I tell my prior authorization team, utilization management team, our system and care team, I tell everyone, you have more of that [member] interaction. You tell me [where the gaps are]*.” Similarly, a vice president of behavioral health reported: “Care management, where they're trying to refer someone to stabilize their health into that system and they're having a struggle getting in … that's where we would feel [access gaps] the most.”

Plans also reported using other metrics to understand access gaps, including tracking the frequency of out-of-state service use, out-of-network authorizations, and services requiring repeated single-case authorizations. Other metrics included tracking the availability of inpatient behavioral health beds, quality measures like follow-up after hospitalization and emergency room visits, length of wait lists for specific services like assertive community treatment, and service utilization per member by provider. MCOs also reported conducting internal secret shopper assessments to evaluate appointment availability, although one plan cited specific challenges with these evaluations: “*The majority of the time, the behavioral providers are the ones that answer their own phones … the time [to] get back and if they get back to you, is the question.*”

### Theme 3: limited corrective actions were available when access gaps were identified

If an access gap was identified, corrective actions were usually limited. One MCO reported having to file a quarterly corrective action plan with their state to report persistent access limitations. However, because of underlying provider shortages, no solutions were readily available. In states where corrective actions were enforced, they were perceived primarily as punitive, reducing the incentives for plans to identify and report true gaps in their networks. For example, one plan described being “backed in” to contracting with “unqualified” providers to meet minimum time and distance standards. Another MCO reported that a significant detected access gap would mandate an enrollment reduction: “*[Members] would go on fee-for-service. I don't think it's in the best interest of that community for that to happen*.*”* Finally, a national carrier with multiple coverage areas reported that an inability to meet network adequacy standards could disincentivize them from entering a new market.

MCOs that identified access gaps for specific services or populations (eg, children and adolescents) largely focused internally on provider recruitment, outreach, and retention efforts. Unfortunately, these efforts were of limited utility in areas with underlying provider shortages. In these cases, MCOs reported a variety of strategies to increase capacity, including building primary care capabilities to serve mental health needs; relying on utilizing single-case agreements (ie, a one-time contract between the MCO and a provider permitting out-of-network services); and focusing on more aggressive case management to relieve bottlenecks for more acute or intensive care. Lastly, digital health solutions, including telehealth, were used when appropriate, although some states did not allow telehealth access to count toward network adequacy requirements.

### Theme 4: redesigned network assessments could more meaningfully inform access gaps

MCOs offered several considerations to better capture access gaps within their networks and use this information in meaningful ways. Participants identified a need for more surveys and needs assessments grounded in member experiences. They also expressed the desire to focus on accessibility and patient-facing outcomes over traditional time and distance standards. Finally, MCOs favored tracking network performance through quality metrics and provider service characteristics, including cultural competency, language ability, population focus, or specialized skillsets. Along these lines, a senior VP of provider network operations said: “*Really understanding the community's needs…our communities have really different needs across [counties] based on school systems, the corrections system … so figuring out a way to weave that into network adequacy is a big challenge*.”

### Theme 5: MCOs report challenges in maintaining accurate provider directories

Participants highlighted serious logistical difficulties in maintaining accurate provider directories, whether these were ultimately member-facing or submitted to insurance regulators. Data collection, validation, and maintenance of provider directories were often conducted manually and internally with limited staff, though some MCOs used external third-party vendors. Challenges included frequent provider turnover and practice changes, delays or errors in provider reporting, and human errors in data entry. Participants felt that these factors all but ensured that provider directories were out-of-date as soon as data were collected. Additionally, MCOs reported that the types of information available in provider directories were not always most useful to members: “*We will list on our provider directory that Susan Smith is a therapist. We don't know if she focuses in addiction therapy, marriage and family counseling, autism? We don’t have the data to help members make informed decisions*.”

Due in part to regulatory requirements, MCOs also reported efforts to mitigate inaccuracies by auditing their provider directories, conducting spot checks for quality assurance, and utilizing member complaints to update identified errors. However, there was little accountability for ensuring provider directory accuracy. A director of provider network strategy highlighted: “*There may be a diffusion of responsibility here where we might say, “Well, that's the state's job.” They may say, “Well, we send it to the MCOs, that's their job.”* Some participants advocated for centralized provider directories that could digitalize data collection processes and reduce administrative burdens, though there could be tradeoffs. One MCO in a state with such a centralized provider directory reported that this restructuring made it more difficult to address errors nimbly: “*There are some providers [in the state database] … and they speak 87 languages … I don't know that there's a concerted effort and adequate resource dedication to accuracy.”*

## Discussion

Behavioral health access challenges are particularly prominent in Medicaid, and state and federal regulators have sought to ensure patient access through network adequacy regulations. To our knowledge, this is the first study to examine MCOs' perceptions of ongoing efforts to monitor access in behavioral health care. Our findings highlight substantial challenges facing health plans within the broader constraints of the delivery system, including workforce shortages and maldistribution. MCOs shared insights into their approaches and limitations in assessing access gaps as well as tensions between regulatory guardrails and organizational responses. These findings align with prior research identifying several challenges in this area, including unclear understandings of what constitutes an adequate network, with wide variation in standards across states and markets;^31,32^ widespread provider directory inaccuracies and inadequate enforcement,^23,25,33^ and fluctuating provider and health plan contracting arrangements that challenge data collection and verification.^34^

While our interviews focused on behavioral health specifically (a field that has unique provider supply constraints and high service demand), our findings have key implications for ongoing policy efforts to improve access to Medicaid services more broadly. For example, MCOs provided important insights into well-documented and widespread provider directory inaccuracies that are not limited to behavioral health. Plans' acknowledgement of provider directory inaccuracies was consistent with prior studies suggesting that approximately 50% of provider directory entries have at least one inaccuracy, including incorrect contact information, specialty, and insurance network status.^25,35-37^ In addition, studies suggest that many in-network physicians may not actually deliver care to Medicaid enrollees,^18,24^ raising questions about provider capacity, which is not captured by directory data.

Provider directories are intended to help consumers select health plans while also serving as a tool for state regulators to evaluate network adequacy. Our findings reinforce broader concerns that provider directories, as currently implemented, are insufficient to meet these needs. These challenges may be particularly acute in behavioral health, where solo and small group practices often operate with limited administrative capacity to keep directories updated and respond to health plan data requests^11,12^ More real-time and simplified processes, with adequate support structures, are needed to help ensure that provider directory data can be meaningfully used.

Importantly, network adequacy standards are viewed as one expansive but potentially imprecise lever with which to hold MCOs accountable for their enrollees' ability to access care. Medicaid regulations span from minimum provider-to-enrollee ratios, to distinct travel time and distance requirements and minimum wait time standards.^31,38^ However, current standards have not been associated with improved access to care, patient experience, or outcomes^39,40^ and they often do not align well with consumer preferences.^41^ Our findings highlight a number of important reasons why this may be the case, including misalignment between collected measures and access goals, leading to data that inadequately reflects member needs, timely access to care, and/or quality of services. As a result, participants reported using member grievances as well as utilization and case management reports to identify critical gaps in behavioral health access—all poorly captured by state standards.

Because of punitive or ineffective corrective action plans, MCOs described little incentive to report these true access gaps to regulators. At the extreme, MCOs identified specific behavioral changes—including contracting with poorer quality providers or avoiding entry into certain markets—in order to minimize punitive risk. While little empirical evidence is available on the effects of network adequacy standards on MCO behaviors, these findings highlight important tradeoffs for policymakers to consider. Taken together, these findings highlight key gaps between policy intentions and efficacy, with implications that extend far beyond behavioral health.

Finally, in an effort to strengthen network adequacy, the Centers for Medicare and Medicaid Services (CMS) released new Medicaid managed care rules in April 2024, calling for maximum appointment wait time standards, implementing audits of managed care compliance, and public transparency of this performance data.^42^ In the context of our findings, these efforts introduce important considerations. Network adequacy regulations may provide significant value that MCO participants may not see on the ground, including curtailing prohibited actions like adverse selection or inappropriate service denials intended to maximize profits. At the same time, our findings reveal that current regulations do not address the most pressing access problems that MCOs are facing. If the role of regulation is also to help ensure better access to care, then there may be opportunities to support MCOs more broadly through network adequacy functions. Our interviews revealed an appetite from MCOs to improve current monitoring efforts, as well as a desire to help inform policy development and broader solutions to critical behavioral health access challenges.

## Conclusion

Improving access to behavioral health providers is essential for Medicaid enrollees, yet assessments of current regulatory efforts raise questions about whether existing approaches are effectively addressing this need. While network adequacy standards are intended to ensure consumer access, MCOs question their ability to reflect the realities of provider availability, capacity, and patient needs. New CMS regulations may offer a step forward, but success may depend on addressing underlying issues like workforce constraints, reimbursement rate differentials between Medicaid and other programs, and the potential for misaligned incentives among MCOs.

## Supplementary Material

qxaf049_Supplementary_Data
